# Central retinal artery occlusion preceding hemispheric ischemic stroke treated with mechanical thrombectomy: A case report

**DOI:** 10.1097/MD.0000000000051229

**Published:** 2026-10-23

**Authors:** XiaoYan Wen, Baoyi Guo, Xue Zeng, Zerui Xiang, Zhibo Deng, Zhengzhou Yuan

**Affiliations:** a Department of Neurology, Neijiang Dongxing District People’s Hospital, Neijiang, China; b Student, Clinical Medical School, Southwest Medical University, Luzhou, China; c Department of Neurology, The affiliated Hospital of Southwest Medical University, Luzhou, China.

**Keywords:** acute ischemic stroke, case report, central retinal artery occlusion, internal carotid artery occlusion, mechanical thrombectomy

## Abstract

**Rationale:**

Central retinal artery occlusion (CRAO) is a form of ischemic stroke that may signal underlying cerebrovascular or cardiac disease.

**Patient concerns:**

A 52-year-old man developed sudden, painless vision loss in the right eye. Nine days later, he presented with left-sided weakness and speech difficulty, with a National Institutes of Health Stroke Scale (NIHSS) score of 8. His medical history included dilated cardiomyopathy, and electrocardiography revealed atrial fibrillation.

**Diagnoses:**

Ophthalmologic examination and fluorescein angiography supported right CRAO. Computed tomography (CT) angiography at neurological presentation showed right internal carotid artery occlusion from its origin. CT perfusion demonstrated estimated infarct core and penumbral volumes of 37 and 186 mL, respectively.

**Interventions:**

Emergency mechanical thrombectomy with aspiration and adjunctive balloon angioplasty achieved expanded Thrombolysis in Cerebral Infarction grade 2c reperfusion.

**Outcomes:**

The NIHSS score improved to 2 at discharge, 7 days after admission. At 3 months, the modified Rankin Scale score was 1, although right-eye visual loss persisted.

**Lessons:**

CRAO warrants prompt cerebrovascular and cardiac evaluation, with reassessment when new neurological deficits develop. The nine-day interval between retinal symptoms and thrombectomy does not establish the duration of carotid occlusion or cerebral ischemia. The favorable individual outcome does not establish the efficacy of thrombectomy beyond 24 hours.

## 1. Introduction

Acute ischemic stroke (AIS) is a leading cause of disability and mortality worldwide. It is defined as the sudden onset of neurological dysfunction caused by a focal infarction in the brain, spinal cord, or retina. Although cerebral infarctions are well-recognized in stroke protocols, retinal ischemia, particularly central retinal artery occlusion (CRAO), is often overlooked as an equivalent vascular emergency. The American Heart Association/American Stroke Association (AHA/ASA) guidelines emphasize that CRAO should be regarded as an ocular manifestation of stroke, warranting the same degree of urgency and diagnostic evaluation.^[1]^

CRAO presents with sudden, painless monocular vision loss and is most commonly caused by embolic or thrombotic occlusion of the central retinal artery or its branches. Although often managed within ophthalmology, accumulating evidence suggests that CRAO is frequently associated with systemic vascular pathology. Studies indicate that 15 to 30% of patients with CRAO develop a subsequent cerebral infarction, often within days of the initial ocular event.^[2]^ Magnetic resonance imaging (MRI) revealed silent infarctions in approximately one-third of CRAO cases. These findings underscore the critical need for early neurovascular evaluation of patients with acute retinal ischemia.

Internal carotid artery occlusion (ICAO) is an underlying cause of CRAO that may lead to ophthalmic artery embolism and CRAO as early symptoms. ICAO is a severe and potentially fatal condition that often results in large-territory stroke with devastating outcomes. In some cases, CRAO may be the only symptom observed during the early stages of ICAO, delaying timely diagnosis and intervention. The misclassification of CRAO as an isolated ophthalmic issue can result in missed opportunities for systemic stroke prevention and reperfusion therapy.

Mechanical thrombectomy (MT) has revolutionized the treatment of AIS caused by large-vessel occlusion (LVO), with multiple randomized controlled trials establishing its benefits within a 6-hour window. Subsequent trials have demonstrated that, with advanced perfusion imaging, the treatment window can be safely extended for up to 24 hours in carefully selected patients.^[3,4]^ However, the role of MT beyond 24 hours remains controversial and is currently supported by limited observational data and case reports. Ultra-late thrombectomy, defined as MT performed > 24 hours after symptom onset, has rarely been explored in the context of CRAO-associated ICAO.

In this report, we present the case of a 52-year-old man who initially presented with CRAO and developed hemispheric stroke symptoms associated with ICAO 9 days later. The patient underwent successful mechanical thrombectomy, followed by a favorable neurological outcome. The interval between the ocular and hemispheric events does not establish the onset or duration of ICAO or cerebral ischemia. This case highlights CRAO as a warning sign of potentially serious underlying cerebrovascular disease and reinforces the importance of prompt vascular and cardiac evaluation, together with urgent reassessment when new neurological deficits develop.

## 2. Case report

A 52-year-old, right-handed man presented to the emergency department with a sudden onset of left-sided weakness and speech difficulty. His initial National Institutes of Health Stroke Scale (NIHSS) score was 8. Nine days prior, he experienced a sudden, painless loss of vision in his right eye. At that time, he visited an ophthalmology clinic, where fundus examination revealed retinal pallor with a characteristic “cherry-red spot” in the macula. Fluorescein angiography showed delayed central retinal artery filling, and optical coherence tomography (OCT) demonstrated macular edema and increased reflectivity (Fig. 1A–E). A diagnosis of central retinal artery occlusion (CRAO) was made. He received topical intraocular pressure-lowering agents and retrobulbar tolazoline injections, but visual loss persisted. No systemic neurological workup was performed, and the patient was discharged the following day.

**Figure 1.**
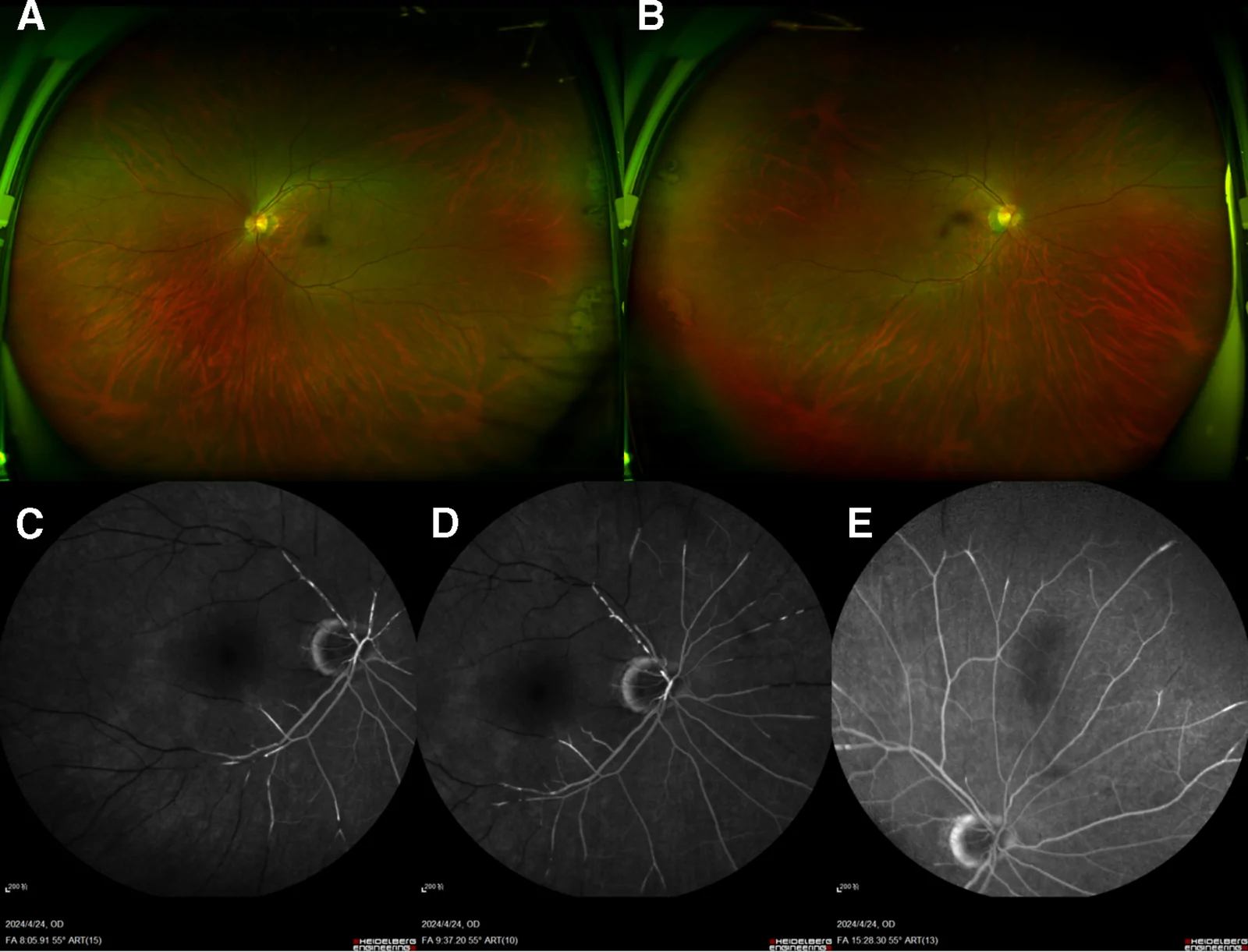
Multimodal imaging of central retinal artery occlusion in the right eye. (A–F) Fundus fluorescein angiography (FFA) and scanning laser ophthalmoscopy (SLO) reveal a pale, edematous retina with a macular “cherry-red spot.” FFA shows delayed arterial filling and non-perfusion in the superior temporal, inferior temporal, and superior nasal branch arterioles during the early phase, followed by segmental hyperfluorescence in the mid-to-late phases. (G, H) Optical coherence tomography (OCT) demonstrates macular edema, retinal thickening, and inner retinal hyperreflectivity.

At the time of neurological presentation, 9 days later, he exhibited left hemiparesis and expressive aphasia. Vital signs included a blood pressure of 76/51 mm Hg and a heart rate of 122 bpm. Electrocardiography revealed atrial fibrillation (AF). His medical history included hypertension, type 2 diabetes mellitus, and dilated cardiomyopathy (DCM). He had undergone pacemaker implantation 2 years earlier. Laboratory evaluation revealed elevated N-terminal pro-brain natriuretic peptide levels (1683 pg/mL), mild hyperglycemia, and a normal coagulation profile. Non-contrast computed tomography (CT) of the head showed subtle loss of gray-white matter differentiation in the right cerebral hemisphere with an ASPECTS score of 9.

Computed tomography angiography (CTA) demonstrated complete occlusion of the right internal carotid artery (ICA) from its origin. CT perfusion (CTP) imaging revealed a substantial mismatch between a large ischemic penumbra (186 mL) and a small infarct core (37 mL), indicating significant salvageable brain tissue. Cervical Doppler ultrasonography confirmed the absence of right ICA flow (Fig. 2A–G). In view of the patient’s acute neurological deterioration, favorable perfusion profile, and absence of hemorrhagic transformation, he was deemed a candidate for emergency endovascular therapy.

**Figure 2.**
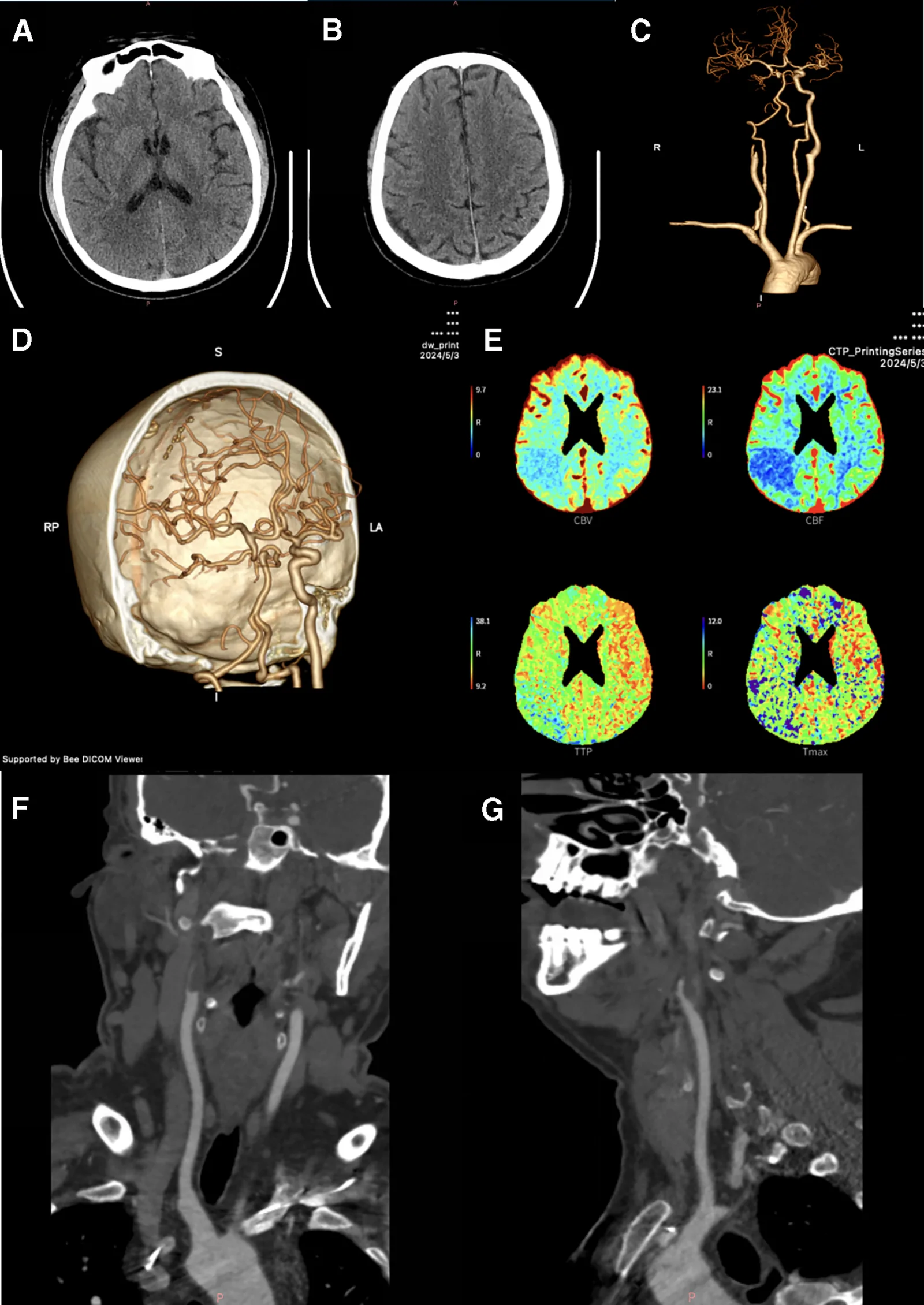
Preoperative imaging for right internal carotid artery occlusion. (A, B) Non-contrast CT brain (ASPECTS 9) shows minimal early ischemic change. (C, D) CTA reconstruction visualizes the right ICA occlusion. (E) CTP demonstrates a significant ischemic penumbra (outlined) in the right temporal and parietal lobes. (F, G) CTA-MIP image confirms the site of occlusion.

### 2.1. Endovascular procedure

Under local anesthesia and conscious sedation, an 8F balloon guiding catheter (Tongqiao Medical, China) was advanced via the right femoral artery to the cervical segment of the right ICA. A Rebar-18 microcatheter (Medtronic, USA) was advanced over a 0.014-inch microwire (Stryker, USA) into the right middle cerebral artery (MCA) M1 segment. A 5 × 30 mm stent retriever (Tongqiao Medical, China) was deployed at the proximal site of occlusion. After three passes of aspiration and stent retrieval, partial recanalization was achieved. The residual thrombus in the cavernous segment prompted additional balloon angioplasty. Final digital subtraction angiography (DSA) demonstrated successful reperfusion of the right ICA and its downstream branches, including the MCA and the anterior cerebral artery (Fig. 3A–J), with expanded thrombolysis in cerebral infarction (eTICI) grade 2c. The ophthalmic artery did not show contrast opacification post-recanalization, suggesting irreversible occlusion.

**Figure 3.**
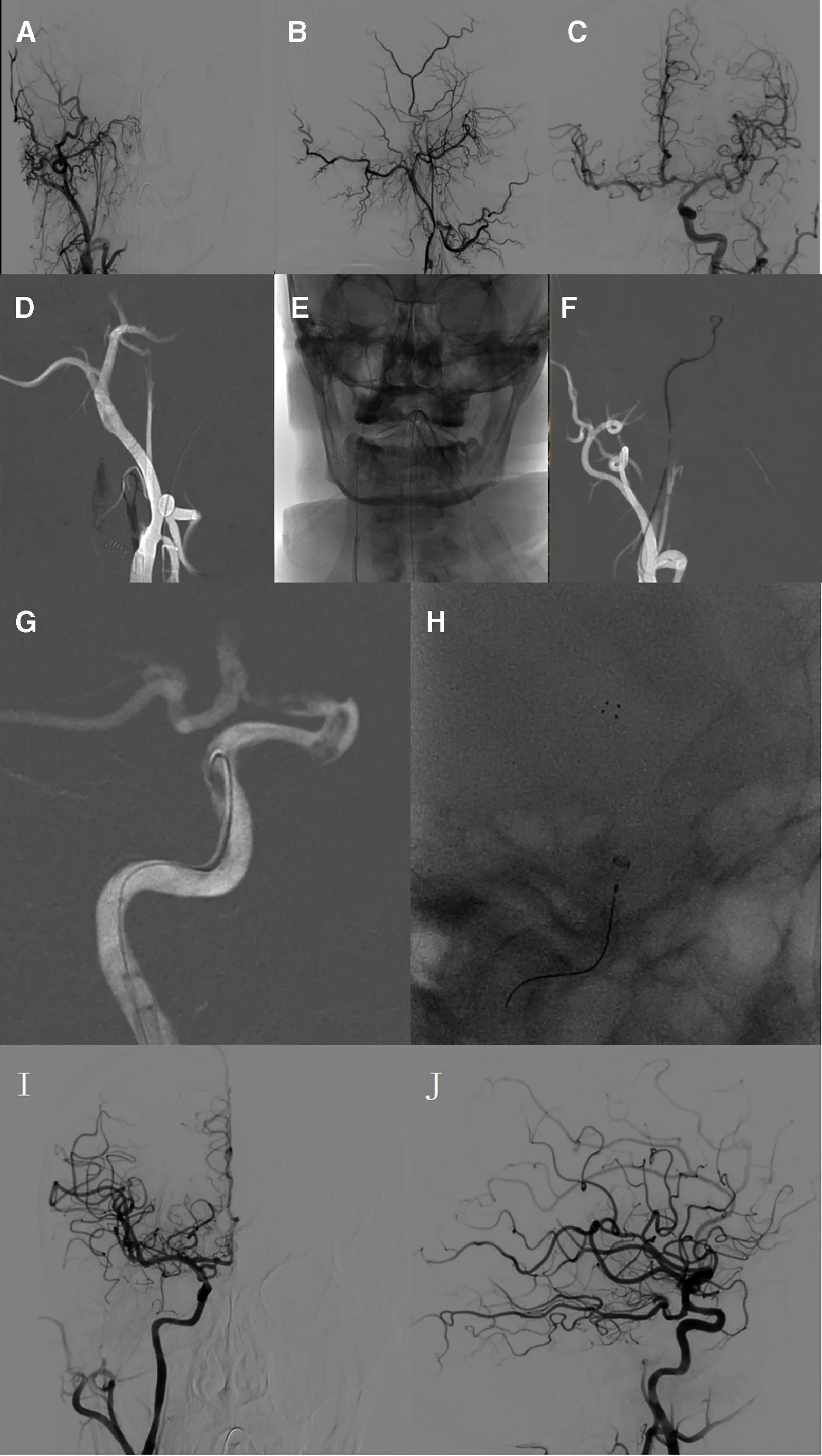
Endovascular recanalization of an occluded right internal carotid artery (ICA). (A–C) Digital subtraction angiography shows occlusion at the right ICA origin, with collateral filling of the right middle cerebral artery via the patent anterior communicating artery from the left side. (D–F) Thrombus aspiration was performed. (G, H) Due to residual thrombus, a thrombectomy stent was deployed. (I, J) Final angiography achieved successful recanalization (eTICI grade 2C) of the right ICA. The ophthalmic artery was not visualized post-procedure.

### 2.2. Postoperative course

Head CT performed immediately after thrombectomy showed no evidence of hemorrhagic transformation but revealed slight cortical hypodensities in the right temporoparietal sulci, attributed to contrast extravasation. Rivaroxaban 10 mg once daily was initiated on postoperative day 3 for secondary stroke prevention. The patient’s neurological symptoms progressively improved, with the NIHSS score decreasing from 8 to 2 at discharge, 7 days after admission. No complications such as groin hematoma, renal dysfunction, or recurrent embolic events were noted.

At the 3-month follow-up, telephone assessment indicated a modified Rankin Scale (mRS) score of 1, reflecting mild residual language difficulty but with functional independence. The patient’s visual loss in the right eye remained unchanged, consistent with the irreversible nature of prolonged retinal ischemia.

## 3. Discussion

We describe the case of a patient who initially presented with central retinal artery occlusion (CRAO) and developed acute neurological deficits secondary to internal carotid artery occlusion (ICAO). Despite the significant delay from symptom onset to treatment, the patient underwent successful mechanical thrombectomy (MT), with marked neurological improvement and favorable functional outcomes. This case highlights CRAO as a warning sign of potentially serious underlying cerebrovascular disease and the importance of prompt cerebrovascular evaluation in patients with acute monocular visual loss.

CRAO is increasingly being recognized as an ophthalmologic emergency and a form of acute ischemic stroke (AIS). It typically presents with sudden, painless monocular vision loss and is caused by embolic or thrombotic occlusion of the central retinal or ophthalmic arteries. Ophthalmoscopy usually reveals retinal whitening and cherry-red spots in the fovea. Although neuroimaging is not mandatory for diagnosing CRAO, diffusion-weighted MRI can detect retinal diffusion restriction with a sensitivity of 62.6 to 75% within the first week, which correlates with the extent of ischemic retinal edema seen on OCT^[2,5]^ Nevertheless, establishing the ocular diagnosis does not replace the need for urgent cerebrovascular evaluation.

The visual prognosis for CRAO is generally poor. Less than 20% of patients recover functional vision, largely depending on the duration and extent of retinal ischemia and the adequacy of collateral circulation.^[6]^ Epidemiologically, CRAO occurs at a rate of approximately 1.8 to 2.5 per 100,000 individuals annually and is strongly associated with vascular risk factors such as hypertension, diabetes, dyslipidemia, obesity, and smoking.^[5]^ Numerous studies have highlighted the systemic implications of CRAO. In a 6.4-year follow-up study of patients with acute retinal arterial occlusion, 7.4% experienced major cardiovascular events, two-thirds of which were ischemic strokes.^[7]^ Furthermore, approximately 30% of patients with CRAO show evidence of silent cerebral infarction on MRI.^[8]^These findings support urgent neurological assessment and brain imaging after CRAO, and the early identification of underlying large-vessel disease is critical.

ICAO, in particular, has been linked to both CRAO and AIS. The pathophysiology often involves atherosclerotic narrowing or embolic occlusion of the internal carotid artery, with subsequent downstream embolization of the ophthalmic or retinal arteries. In our case, carotid-related embolism or impaired perfusion could have contributed to the retinal and cerebral ischemic events. However, atrial fibrillation and dilated cardiomyopathy also raise the possibility of cardioembolism, and the available evidence does not establish a single embolic source or mechanism.

Patient selection for MT should integrate the severity of the presenting neurological deficits, the timing of the cerebral ischemic event, prestroke functional status, infarct extent, potentially salvageable tissue, collateral circulation, and vascular anatomy. DAWN demonstrated benefit in selected patients 6 to 24 hours after last known well using age-adjusted clinical–core mismatch, whereas DEFUSE 3 used perfusion-based selection at 6 to 16 hours, including an NIHSS score ≥ 6, an infarct core < 70 mL, a mismatch ratio ≥ 1.8, and a mismatch volume ≥ 15 mL.^[3,4]^ MR CLEAN-LATE subsequently supported collateral-based selection on CTA in the 6 to 24-hour window.^[9]^ In our patient, an NIHSS score of 8, an ASPECTS of 9, and a reported infarct core of 37 mL with perfusion mismatch supported consideration of endovascular treatment. However, the NIHSS score was below the DAWN eligibility threshold, and these findings alone do not establish complete eligibility under a specific late-window trial. The location and extent of ICAO, associated intracranial occlusion, and any residual stenosis also require assessment, particularly when adjunctive angioplasty is considered.

Potential benefits must be weighed against intracranial hemorrhage, distal embolization, arterial dissection or perforation, reperfusion injury, and access-site complications. The ANGEL-REBOOT randomized trial illustrates the additional risks of rescue procedures: among patients with unsuccessful recanalization or severe residual stenosis after thrombectomy, bailout intracranial angioplasty or stenting was associated with more symptomatic intracranial hemorrhage (5% vs 1%) and procedure-related arterial dissection (14% vs 3%) than standard therapy.^[10]^ These findings emphasize the need for a clear anatomical and procedural indication for adjunctive angioplasty; they should not be interpreted as risk estimates applicable to all patients undergoing MT. In our patient, 3 thrombectomy passes and adjunctive balloon angioplasty made assessment of the residual lesion and potential vessel injury particularly relevant. AF and dilated cardiomyopathy also increased the importance of evaluating cardiac function, hemodynamic stability, and the periprocedural antithrombotic strategy.

Beyond 24 hours, the retrospective SELECT Late study reported an association between MT and functional independence, but symptomatic intracranial hemorrhage occurred more frequently in the MT group than in the medical-management group (10.1% vs 1.8%). Selection bias and residual confounding limit causal interpretation of these findings.^[11]^ Prospective efforts to clarify patient selection and safety include the published LATE-MT protocol and the currently recruiting SELECT LATE randomized trial (NCT07115940), both addressing treatment in the 24 to 72-hour window.^[12]^ Their assessment of functional outcomes alongside hemorrhagic and other safety outcomes reflects the need to establish the benefit–risk balance in defined populations. Trial protocols represent research eligibility criteria and should not be treated as established treatment indications. In the present case, the interval from CRAO to MT cannot determine the cerebral treatment window, and the favorable individual outcome does not establish the efficacy or safety of thrombectomy beyond 24 hours.

Collateral circulation may help preserve viable cerebral tissue, but its presence does not establish the duration of arterial occlusion. In our patient, CT perfusion imaging performed 9 days after the initial CRAO showed a large estimated penumbra (186 mL) with a relatively small estimated infarct core (37 mL), suggesting potentially salvageable tissue at the time of assessment. These findings do not establish that ICAO had been continuously present for 9 days or that the same penumbra persisted throughout this interval. Because the hemispheric neurological deficits developed later, the interval from CRAO to thrombectomy cannot be equated with the duration of ICAO or cerebral ischemia. This highlights the importance of individualized decision-making that integrates perfusion imaging with the clinical presentation and timing of the cerebral ischemic event.

Despite successful recanalization, the patient’s vision in the right eye did not recover, likely because of irreversible retinal infarction sustained during the initial CRAO episode. This underscores the limited time window for retinal salvage, which is generally believed to be within 4 to 6 hours. Nevertheless, neurological improvement and good functional recovery (mRS 1 at 3 months) represent a favorable individual outcome after MT, but do not establish the efficacy of treatment beyond 24 hours. Visual and neurological outcomes should therefore be interpreted separately.

### 3.1. Limitations

This case report has several limitations. First, the available vascular imaging does not establish the precise onset or uninterrupted duration of ICAO, and serial perfusion imaging was unavailable to characterize tissue viability during the interval between the retinal and cerebral events. Second, the available angiographic and ophthalmic follow-up data do not establish whether retinal reperfusion occurred. Therefore, the effects of thrombectomy on retinal reperfusion remain unclear. Third, the mechanism linking the retinal and cerebral ischemic events remains uncertain. Both carotid-related mechanisms and cardioembolism are plausible in this patient. The available evidence does not establish a single embolic source. Additionally, as a single case, this report cannot establish causal inference or generalizability, or demonstrate the efficacy or safety of MT beyond 24 hours, but it serves as a hypothesis-generating observation.

## 4. Conclusion

This case highlights central retinal artery occlusion (CRAO) as a potential warning sign of serious underlying cerebrovascular disease, particularly internal carotid artery occlusion (ICAO). Sudden painless monocular vision loss should prompt urgent neurovascular evaluation, including cervical vascular imaging, together with assessment for cardiac sources of embolism. In this patient, MT was followed by a favorable neurological outcome, while visual loss persisted. However, the nine-day interval from CRAO to thrombectomy does not establish the duration of ICAO or cerebral ischemia. This case emphasizes the importance of prompt evaluation and individualized treatment decisions that integrate the presenting neurological event, contemporaneous imaging, and procedural risks; it cannot establish the efficacy or safety of thrombectomy beyond 24 hours. Future prospective studies are essential to better define the patient selection criteria, safety, and long-term efficacy of ultra-late thrombectomy.

## Acknowledgments

We sincerely thank the patient for consenting to the publication of this case report.

## Author contributions

**Investigation:** XiaoYan Wen, Baoyi Guo.

**Writing – original draft:** XiaoYan Wen, Baoyi Guo.

**Resources:** Zhengzhou Yuan, Xue Zeng, Zerui Xiang.

**Writing – review & editing:** Zhibo Deng, Zhengzhou Yuan.

**Conceptualization:** Zhengzhou Yuan.

**Supervision:** Zhengzhou Yuan.
